# Comparison of open reduction and fixation with hook plate and modified closed reduction and fixation with tightrope loop plate for treatment of rockwood type III acromioclavicular joint dislocation

**DOI:** 10.1186/s12891-022-05261-5

**Published:** 2022-03-29

**Authors:** Song Liu, Chunxia Li, Zhaohui Song, Xiaodong Bai, Haotian Wu

**Affiliations:** 1 Department of Orthopaedic Surgery, the 3rd Hospital of Hebei Medical University, Shijiazhuang, 050051 Hebei People’s Republic of China; 2 Key Laboratory of Biomechanics of Hebei Province, Shijiazhuang, 050051 Hebei People’s Republic of China; 3 Department of Imaging Medicine, General Hospital of Inner Mongolia Autonomous Region, Hohhot, 010017 Inner Mongolia People’s Republic of China

**Keywords:** Acromioclavicular joint dislocation, Minimal invasive surgery, Tightrope loop plate fixation, Hook plate fixation, Clinical results

## Abstract

**Objective:**

To compare the outcomes of open reduction and hook plate fixation (ORHPF) and modified TightRope loop plate fixation (MTRLPF) in the treatment of Rockwood type III acromioclavicular joint dislocation.

**Methods:**

This was a retrospective study. Data on 71 patients with Rockwood type III acromioclavicular joint dislocation who underwent either ORHPF (*n* = 39) or MTRLPF (*n* = 32) between January 2016 and October 2019 were extracted and analyzed. Baseline data at injury were compared to evaluate the balance. The disabilities of the arm, shoulder, and hand (DASH) score, Constant-Murley score and visual analog scores (VAS) score at 1 month, 3 months, 6 months and 12 months after operation were compared; further, at 12 months coracoclavicular distance and related complications were evaluated and compared.

**Results:**

Both groups did not differ for any baseline data. At 1 and 3 months after operation, MTRLPF group exhibited a significantly better performance than the ORHPF group in VAS (1 month: 2.4 ± 1.8 vs 3.0 ± 1.7; 3 months: 1.2 ± 1.4 vs 1.8 ± 1.6), Constant-Murley (1 month: 75.2 ± 11.2 vs 63.8 ± 13.7; 3 months: 81.4 ± 9.8 vs 75.8 ± 10.6), DASH (1 month: 33.6 ± 6.8 vs 40.6 ± 6.1; 3 months: 21.2 ± 7.4 vs 25.6 ± 6.6). At 6 months, only Constant-Murley remained marginally significant (*p* = 0.048). At 12 months, no statistical difference was observed for any outcome variable (all *P* > 0.05 for VAS, Constant-Murley and DASH), coracoclavicular distance (12.7 ± 1.6 mm vs 12.2 ± 1.6 mm; *P* = 0.374), or overall complication rate (*P* = 0.763).

**Conclusions:**

For Rockwood type III acromioclavicular joint dislocation, both methods can achieve satisfactory 1-year results, but modified minimally invasive TightRope treatment is more advantageous in early functional recovery at 1 and 3-month follow-ups.

## Introduction

Acromioclavicular joint dislocation represents a common injury in emergency and orthopaedics department, with an incidence of 5.5/100,000 person-years [1], and accounts for 9%-12% of shoulder traumatic cases [2, 3]. In most cases, acromioclavicular joint dislocation occurs in competitive sports, in which shoulder lands during falls and forces acts directly on the shoulder peak and pushes the scapula downward, with resultant acromioclavicular ligament or even combined coracoclavicular ligament injury or rupture. At present, there is a generally accepted principle for the treatment of acromioclavicular joint dislocation, that is, conservative treatment for Rockwood type I-II dislocation and surgical treatment for type IV-VI [4]. However, there is still controversy in the treatment of type III dislocation even up to 150 treatments have been proposed [4]. Conservative treatment of type III dislocation has been reported to achieve favorable shoulder joint functional recovery, but it is limited in use due to difficulty in reducing the acromioclavicular joint; furthermore, the residual deformity may produce cumulative changes in the movement of the shoulder joint, and a substantial group of patients experiencing conservative treatment were dissatisfied and underwent delayed surgical reconstruction [5].

In contrast, surgery is preferably used for type III acromioclavicular joint dislocation. Among surgical methods, open reduction and hook plate fixation (ORHPF) is most widely used with reported favorable clinical outcomes, but it is susceptible to complications, such as subacromial impingement, bone erosion, persistent pain and so on [2, 6, 7]. In recent decade, minimally invasive treatment, e.g. arthroscope-assisted TightRope loop plate fixation, has become admired, which can not only meet the aesthetic requirements, but also can achieve faster function recovery than the traditional method [2]. However, this method is technically more demanding, and deviation of drilling position may lead to surgical failure. Furthermore, arthroscopy has not been popularized in primary or secondary hospitals in areas with relatively poor economic or medical conditions. Given that, we modified the TightRope loop plating technique, using the closed reduction via a minimally invasive incision, which simplified the surgical procedure and showed better preliminary results than conservative method and similar 1-year results as ORHPF [6].

In this study, we extended the study window to include more eligible subjects. Considering the obvious advantages of minimal invasiveness of modified TightRope loop plate fixation (MTRLPF), we hypothesize that MTRLPF could provide more favorable early functional recovery than did ORHPF for treatment of Rockwood type III acromioclavicular joint dislocation.

## Methods

This retrospective study was approved by the institutional review board of the Third Hospital of Hebei Medical University and obtained all the participants' written informed consent before its commencement. We reported this study in accordance with the Helsinki Declaration and following the Strengthening the Reporting of Cohort Studies in Surgery (STROCSS).

### Inclusion and exclusion criteria

The inclusion criteria were: age of 18 to 60 years, definite radiographic diagnosis of isolated Rockwood type III acromioclavicular joint dislocation, time from injury to operation < 2 weeks, and patients with complete at least 12-month follow-up assessments. The exclusion criteria were: age outside the range, open jury, old injury (≥ 2 weeks since injury), injury caused by other diseases (tendinitis, metabolic, et al.), concurrent shoulder osteoarthritis, arthropathy or any fracture, any previous operation of the injured limb, or incomplete data or follow-up < 12 months.

### Surgical technique

#### Modified tightrope loop plate fixation (MTRLPF)

**Fig. 1:**
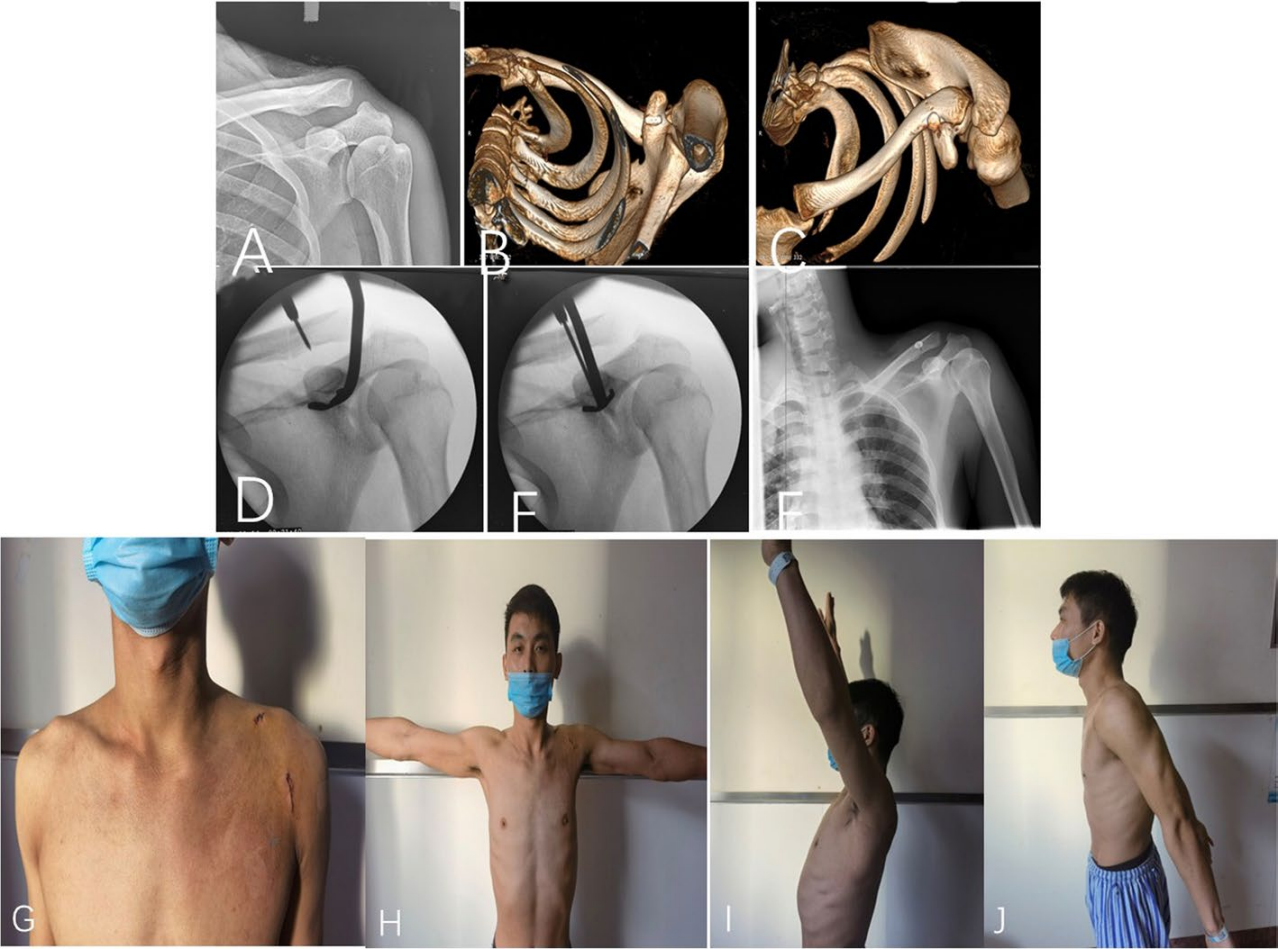
**A-F** Depict the operative procedure process of MTRLPF in a 29-year male patient, who had Rockwood type III acromioclavicular joint dislocation at his left shoulder due to the fall from standing height. **A**-**C** presents the preoperative radiograph (**A**) and three-dimensional reconstruction for the acromioclavicular joint dislocation. **D**-**E** presents the intraoperative operation and F shows the acceptable reduction. **G-J** depict the postoperative shoulder motion range at the 2^nd^ day after operation, showing almost complete recovery

Under general anesthesia or cervical plus brachial plexus block, with patient in a beach-chair position and the affected shoulder up. The projection point of the coracoid was identified under fluoroscopy. A transverse incision 1.0–2.0 cm in length above the projection point was made to the clavicular periosteum to touch the coracoid; then a longitudinal incision 1.5–2.0 cm was made downward at the inferior edge of coracoid, and blunt dissection was preformed straight to the base of the coracoid process. Under the guidance of a self-made guide device, a 2.4-mm diameter guide needle was introduced from the center of the anteroposterior edge of the clavicle to the center of the base of the coracoid process, under fluoroscopy control. A 4.0-mm hollow drill was used to ream, then guide needle was removed and lead device was insert via hollow drill, which was removed before TightRope titanium plate (Arthrex, USA, CFDA: Import 20,173,460,186) was pushed. FiberWire loop above the clavicle was adjusted, fastened and knotted, and then the tail was cut off after fluoroscopy confirmed the satisfactory reduction. The Fig. 1A-F depicts the operative procedure process.

#### Open reduction and hook plate fixation (ORHPF)

Under general anesthesia or cervical plexus plus brachial block, with patient in a beach-chair position, a 6 to 8-cm incision was made along the distal clavicle to the acromion to expose the distal clavicle and the shoulder joint. Under direct vision, shoulder joint was reduced and a hook plate (Tianjin, Zhengtian, CFDA 20,173,464,301) was inserted for fixation. Shoulder joint was passively moved to confirm that there was no acromion impingement, and then acromioclavicular ligament was sutured and repaired. After surgery, the affected shoulder was abducted and fixed in a neutral position to avoid weight-bearing.

#### Postoperative management

Patients in both groups are asked to follow the same postoperative exercise regimen over time. From the 1^st^ day after surgery, active fist clenching and elbow movement was started; from 3^rd^ day, passive pendulum movement exercise of the shoulder joint was started, and affected shoulder was immobilized in abduction when at rest. Passive and active and functional exercises of the shoulder joint were started 2 to 4 weeks after surgery, until the range of motion of the affected shoulder returned to the normal. At 3 months postoperatively, weight-bearing exercises were started and gradually increased. In most cases, the hook plate is removed 6 months after surgery and can be removed early if plate fixation fails or other adverse events have occurred. The TightRope Loop Plate is not to be removed unless otherwise indicated.

#### Evaluation of results and data extraction

All the data of interest were extracted from the patients' hospitalization medical records, operative records and the outpatient follow-up registration at the postoperative 1, 3, 6 and 12 months. The data included demographics (age, sex, and body mass index (BMI)), injury-related variables such as time from injury to operation, involved side, injury mechanism and the coracoclavicular distance before operation, surgery-related variables such as surgical duration, incision length (for MTRLPF, sum of the both incisions length). At each outpatient follow-up at 1, 3, 6, and 12 months postoperatively, patients were asked to indicate their subjective pain degree by the visual analog scores (VAS), and complete both questionnaires, including Constant-Murley shoulder function questionnaire and the disabilities of the arm, shoulder, and hand (DASH) questionnaire, under the explanation and guidance of the doctor.

The DASH questionnaire is a validated questionnaire to evaluate patients’ ability to perform daily activities [8], including 30 items of daily activities, with a potential response score ranging from 0 which represented no disability, to 100 points which represented maximum disability.

Constant-Murley shoulder function scoring questionnaire is a validated questionnaire [9], including 4 parts: perceived pain, activities of daily living, the range of motion of the shoulder joint and the muscle strength, with scoring at highest of 15, 20, 40, and 25 respectively. The total score ranged from 0, which represented the worst, to 100 which represented the normal should function status.

Any complication occurring during operation, reported by the patients or examined at each follow-up, were noted, such as intraoperative nerve or blood vessel damage, coracoid fracture, postoperative surgical site infection, hardware loosening, loss of reduction, persistent shoulder pain, dislocation recurrence et al.

### Statistical analysis

The continuous variables including age, BMI, coracoclavicular distance before and after operation, incision length, intraoperative blood loss, surgical duration, VAS score, DASH and Constant-Murley score were indicated as mean and standard deviation (SD) and the difference for each variable between two groups was evaluated by Student-*t* test or Mann Whitney-U test, on basis of their normality status. Categorical variables including sex, involved side, mechanism, postoperative complications were expressed as number and percentage, and the difference was evaluated by Pearson Chi-square test. Two-tailed *p* value less than 0.05 was considered to be statistically significant. SPSS 24.0 software (IBM corporation, Armonk, NY, USA) was used to perform all the analyses.

## Results

A total of 71 eligible patients with complete at least 12-month follow-up assessments were included, predominantly males (73.2%, 52/71), with a mean age of 40.8 years. Patients underwent MTRLPF or ORHPF 3.2 days after the injury. Patients undergoing MTRLPF and ORHPF did not differ in term of age, sex, BMI any injury-related variables (all *P* > 0.05, Table 1). The coracoclavicular distance was 23.2 mm in MTRLPF group and 22.7 mm in the ORHPF group at admission, not significantly different (*p* = 0.673, Table 1).

**Table 1:** Comparison of demographics and injury-related data between MTRLPF and ORHPF group

Variable	MTRLPF group (*n* = 32)	ORHPF group (*n* = 39)	*P*
**Age**	39.6 ± 8.9	41.8 ± 10.5	0.357
**Sex**			0.814
Male	23 (71.9)	29 (74.4)	
Female	9 (28.1)	10 (25.6)	
**BMI**	24.7 ± 3.2	24.9 ± 2.9	0.479
**Involved side**			0.397
Left	14 (43.8)	21 (53.8)	
Right	18 (56.2)	18 (46.2)	
**Mechanism**			0.416
Fall	9 (28.1)	15 (38.5)	
Traffic injury	6 (18.8)	11 (28.2)	
Sports injury	12 (37.5)	9 (23.1)	
Others or unknown	5 (15.6)	4 (10.3)	
**Coracoclavicular distance at admission**	23.2 ± 3.3	22.7 ± 3.7	0.673
**Time from injury to operation**	3.4 ± 2.1	3.1 ± 2.7	0.595

*MTRLPF* modified tightrope loop plate fixation, *ORHPF* open reduction and hook plate fixation, *BMI*, body mass index

The analyses of procedure-related variables showed significantly reduced surgical duration (42.2 ± 7.6 min vs 54.5 ± 9.4 min), shorter incision length (3.9 ± 0.5 cm vs 9.6 ± 0.7 cm) and less intraoperative blood loss (43.3 ± 14.6 ml vs 83.2 ± 15.3 ml) in MTRLPF group than in ORHPF group.

At the postoperative 1 and 3 months, MTRLPF exhibited the significantly more improvement in term of VAS, Constant-Murley score and the DASH score, than did the ORHPF (all *P* < 0.05). At the 6-month, only Constant-Murley remained statistically significant (*p* = 0.048), although marginally, favoring the MTRLPF. At the 12-month, there was no significant difference between both groups for any the above scoring scale (*p* = 0.443 for VAS, *p* = 0.614 for Constant-Murley score and *p* = 0.704 for DASH score). The coracoclavicular distance at 12-month was 12.2 ± 1.6 mm in the MTRLPF group, not different from that (12.6 ± 1.6 mm) of ORHPF group (Table 2). The Fig. 1G-J depicts the functional range at the 2^nd^ day after MTRLPE, which exhibited the advantage in early recovery.

**Table 2:** Comparison of procedure-related variables and VAS, Constant-Murley and DASH at 1, 3, 6, and 12 months follow-up, between MTRLPF and ORHPF group

Variable	MTRLPF group (*n* = 32)	ORHPF group (*n* = 39)	*P*
**Surgical duration (minutes)**	42.2 ± 7.6	54.5 ± 9.4	0.003
**Incision length (cm)**	3.9 ± 0.5	9.6 ± 0.7	< 0.001
**Intraoperative blood loss (ml)**	43.3 ± 14.6	83.2 ± 15.3	< 0.001
**VAS score**			
Postoperative 1 month	2.4 ± 1.8	3.0 ± 1.7	0.006
Postoperative 3 months	1.2 ± 1.4	1.8 ± 1.6	0.004
Postoperative 6 months	0.7 ± 0.9	0.9 ± 0.6	0.179
Postoperative 12 month	0.3 ± 0.8	0.4 ± 0.8	0.443
**Constant-Murley score**			
Postoperative 1 month	75.2 ± 11.2	63.8 ± 13.7	0.013
Postoperative 3 months	81.4 ± 9.8	75.8 ± 10.6	0.037
Postoperative 6 months	87.6 ± 4.6	84.4 ± 7.3	0.048
Postoperative 12 month	94.8 ± 3.5	94.4 ± 3.2	0.614
**DASH score**			
Postoperative 1 month	33.6 ± 6.8	40.6 ± 6.1	0.008
Postoperative 3 months	21.2 ± 7.4	25.6 ± 6.6	0.040
Postoperative 6 months	13.7 ± 6.3	14.3 ± 5.4	0.202
Postoperative 12 month	4.6 ± 3.9	4.5 ± 3.3	0.704
** Coracoclavicular distance (mm) at 12-month follow-up**	12.2 ± 1.6	12.6 ± 1.6	0.374

*VAS* visual analog scores, *DASH* disabilities of the arm, shoulder, and hand, *MTRLPF* modified tightrope loop plate fixation, *ORHPF* open reduction and hook plate fixation

**Fig. 2:**
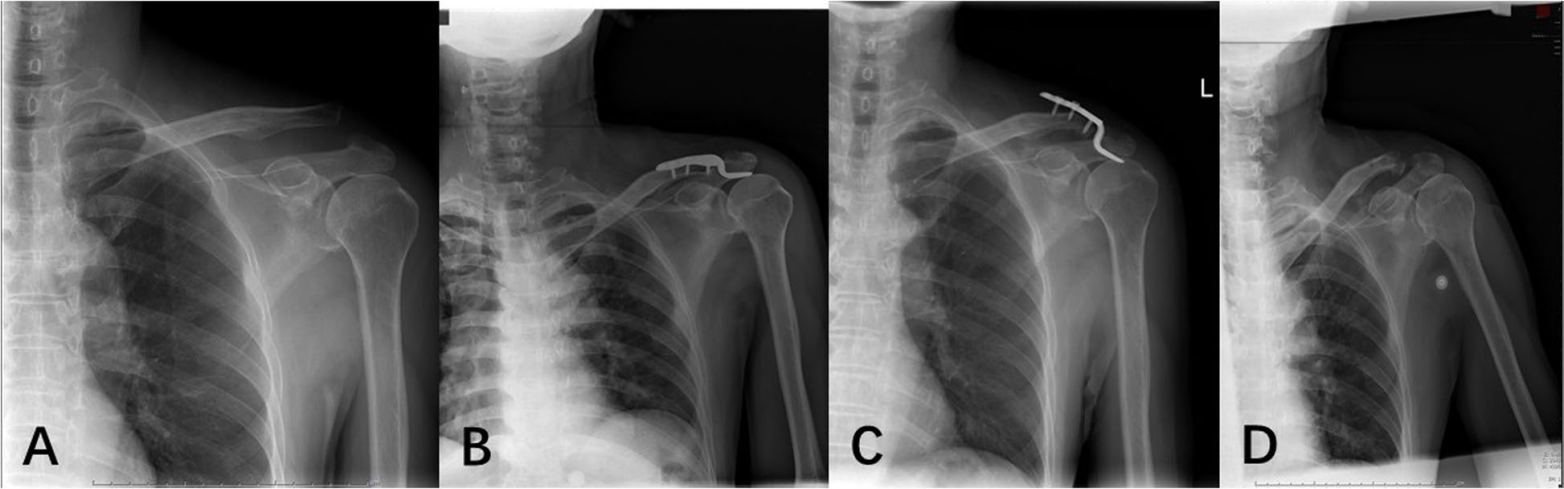
Depict a typical case of complication of distal clavicle fracture at 3 weeks after the open reduction and hook plate fixation (ORHPF) of a Rockwood type III acromioclavicular joint dislocation at a 68-year male patient. Hence, the hardware was removed ahead of the scheduled period

In both groups, no surgical site infection, intraoperative nerve or blood vessel damage, or coracoid fracture was observed. In ORHPF group, 1 patient reported persistent shoulder pain at the 4 months after operation and the symptoms disappeared after the internal hardware was removed ahead of the scheduled period; in 1 patient, subacromial osteolysis was examined on X-rays at 5 months after operation, but the patient did not report obvious symptoms of discomfort and had favorable shoulder function, and hence no special intervention was given; in one patient, distal clavicle fracture developed at the 6^th^ week visit, leading to the fixation failure, and so internal hardware was removed (Fig. 2). In MTRLPF group, 1 patient developed the poor healing of surgical wound; after ruling out the bacterial infections via secretion culture, active dressing changes were prescribed and the surgical wound healed eventually.

## Discussion

In this study, we used a retrospective cohort of relatively small sample size to have demonstrated our hypothesis that for Rockwood type III acromioclavicular joint dislocation, MTRLPF could provide more favorable early functional results than did ORHPF. Meanwhile, compared to ORHPF, MTRLPF was associated with the reduced risk of complications and a better aesthetic effect, supporting its more extensive use in practice.

The biggest advantage of the MTRLPF was to achieve closed reduction of the acromioclavicular joint via 2 small incisions of 1.0 to 2.0 cm in length, with resultant shorter surgical duration and intraoperative blood loss. From the biomechanics view, multiple sets of FiberWires of the TightRope system provided higher mechanical strength than autologous ligament while permitting the flexible fixation in line with bionics, almost completely simulating the motion characteristics of acromioclavicular joint (amphiarthrodial joint, range of 5–7° in physiological state) [10]. Furthermore, two plates of micro profile were implanted above the clavicle bone and under the coracoid, less affecting the motion of the shoulder joint than did the clavicle hook plate. Therefore, it is easy to understand that the patients had a greater pain improvement and quicker functional recovery at the early postoperative period, and we found that this superiority over ORHPF could persist to up to 3 months, even 6 months (although only for Constant-Murley).

The superiority of TightRope loop plate fixation via minimally invasive approach in early functional recovery has also been consistently described in other studies. Boutsiadis et al. [11] described the arthroscopic-assisted TightRope systems for treatment of Chronic acromioclavicular (AC) instability, and suggested that it would have promising excellent radiographic and functional results due to its significant biomechanical and bionics superiority. Yuan et al. [2] compared TightRope loop plate system and clavicle hook plate for treatment of Rockwood type III (23 cases) and IV (9 cases) acromioclavicular joint dislocation, and in their study patients in TightRope group obtained almost completely normal range of motion of shoulder at 4 weeks after operation, and had greatly improvement of VAS than did clavicle hook plate group. Similarly, in a study of 60 cases of Rockwood type III acromioclavicular joint, Zhou et al. [7] reported that arthroscopy-assisted TightRope plate fixation was superior over traditional clavicle hook plate fixation not only in the early functional recovery and VAS improvement at 3-month postoperatively, but also in the 12-month. It is likely that relative to MTRLPF used in the present study, the arthroscopy-assisted reduction might provide a less invasive approach, thus making the difference still be prominent in late results (12-month postoperatively).

From later than 3 months, the superiority of MTRLPF over ORHPF may be gradually diminishing, and most parameters showed non-significant difference, especially at the postoperative 12-month. More than that, even relative to conservative method, these common surgical methods did not exhibit significant superiority in long-term functional results or pain improvement. For example, in the a prospective randomized controlled trial of 60 cases of Type-III and Type-IV acromioclavicular joint dislocations, Murray et al. [5] concluded that open reduction and ThghtRope plating conferred no functional benefits over conservative treatment at 1-year. The similar conclusions have been drawn in other original studies [6, 12, 13] or meta-analysis [14]. This suggested that acromioclavicular or coracoid distance is not necessarily associated with the ultima functional recovery of shoulder joint, although the early functional recovery and pain improvement should be the primary goal of surgical treatments for this subtype of this injury.

It has been well established that, clavicle hook plating is associated with more complications, including excessive reduction, delayed union of the surgical incision, persistent shoulder pain, acromion impingement syndrome, subacromial traumatic arthritis, acromion osteolysis, clavicular hook plate breakage, clavicular stress fracture, and re-dislocation after removal of the hardware [15, 16]. This could be primarily explained by the great biomechanical stiffness of the hook plate that produces the stress concentration under the acromion, and not completely matched profile when selecting the hook plate for fixation, especially during the early period of learning such technique [17]. Additionally, the need for a second operation to remove the internal hardware leads to the extra economic burden. In this study, we only encountered 1 case of shoulder pain and 1 case of subacromial osteolysis, for both of which no special interventions were given. By contrast, in the MTRLPF group only 1 case of poor healing of surgical wound was encountered, which we thought was caused by the excess distraction of the tissues.

This study may suffer from several limitations. First, the retrospective design might have compromised the accuracy in data collection. However, injury-related data were confirmed by the treating surgeon and patients should have a strong memory about their injury, which compensated for the potential risk of recall bias. Second, the small sample and the relatively short follow-up period may not allow detection difference of two treatments in some complications that would develop after a long time, such as subacromial traumatic osteoarthritis, or FiberWire failure. Third, although we obtained the statistically significant difference in clinical outcomes (VAS, DASH, Constant-Murley) at postoperative early period (1 and 3 month), but the maximized absolute difference was 11.4 points for Constant score, which was still less than the minimal clinically important difference between preoperative and postoperative proposed for acromioclavicular joint dislocation by Stein et al. [18]. Therefore, these needs a prospective controlled study to confirm our findings.

In conclusion, this study demonstrated the superiority of MTRLPF over ORHPF in the faster recovery for treatment of Rockwood type III acromioclavicular joint dislocation, while not increasing the risk of postoperative complications. Due to its better biomechanics and bionics design, and the minimal invasive treatment, MTRLPF deserves more consideration in treatment of such injury. Prospective study with controlled design and large sample are warranted to verify our findings and focus on long-term safety assessment of MTRLPE.

## Data Availability

All the data will be available upon motivated request to the corresponding author of the present paper.

## Abbreviations

ORHPF: Open reduction and hook plate fixation
MTRLPF: Modified TightRope loop plate fixation
BMI: Body mass index
VAS: Visual analog scores
DASH: Disabilities of the arm, shoulder, and hand
SD: Standard deviation
